# A Modification of Radical Antegrade Modular Pancreatosplenectomy for Adenocarcinoma of the Left Pancreas: Significance of *En Bloc* Resection Including the Anterior Renal Fascia

**DOI:** 10.1007/s00268-014-2572-5

**Published:** 2014-04-22

**Authors:** Hirohisa Kitagawa, Hidehiro Tajima, Hisatoshi Nakagawara, Isamu Makino, Tomoharu Miyashita, Hirofumi Terakawa, Shinichi Nakanuma, Hironori Hayashi, Hiroyuki Takamura, Tetsuo Ohta

**Affiliations:** Department of Gastroenterologic Surgery, Graduate School of Medical Science, Kanazawa University, 13-1 Takara machi, Kanazawa, 920-8641 Japan

## Abstract

**Background:**

Radical antegrade modular pancreatosplenectomy (RAMPS) has theoretical advantages for curative resection of adenocarcinomas of the left pancreas. The anterior renal fascia is a key structure, and resection planes should run posterior to this fascia. However, it is difficult to delineate this fascia and set a precise dissection plane. We modified RAMPS to achieve such a precise dissection plane with ease.

**Methods:**

After clamping the splenic artery, the third duodenal portion was mobilized from the left to the right to locate the inferior vena cava, which was covered by the anterior renal fascia. Here, the anterior renal fascia was incised while approaching the dissection plane. Dissection then continued cephalad, with this plane along the inferior vena cava, and then turned along the left renal vein at the confluence of the left renal vein toward the renal hilum. At this point, dissection continued along the coronal plane to the superior edge of the pancreas.

**Results:**

Between July 2007 and December 2012, a total of 24 pancreatic adenocarcinoma patients underwent modified RAMPS. Tumor extension beyond the pancreatic parenchyma (T3) and lymph node metastases was confirmed in 17 and 13 cases, respectively. Histologically clear surgical margins were achieved (R0 resection) in 21 patients (88 %). The 5-year overall survival rate was 53 %. Six patients survived for over 5 years without recurrence.

**Conclusions:**

This modification of RAMPS is advantageous for *en bloc* resection while actually including removal of the anterior renal fascia. It is associated with satisfactory survival rates for patients with distal pancreatic carcinomas.

## Introduction

Surgery for pancreatic adenocarcinoma should principally facilitate the achievement of negative resection margins (R0) and *en bloc* dissection of regional lymph nodes, and much effort has been made for these. Pancreatoduodenectomy for carcinomas of the pancreas head has been modified to achieve sufficient resection margins, especially at the pancreatic posterior and uncinate margins [1, 2]. Distal pancreatectomy is the standard procedure for tumors of the left pancreas. However, conventional distal pancreatectomy for ductal carcinomas has traditionally been associated with unfavorable prognoses. Radical antegrade modular pancreatosplenectomy (RAMPS) was designed by Strasberg et al. [3, 4], and was applied for treating carcinomas of the left pancreas, worldwide. RAMPS facilitates good visibility, dissection of N1 nodes, and tumor isolation following early arterial clamping. However, precisely delineating the anterior renal fascia and achieving a precise dissection plane posterior to the pancreas is difficult. We modified RAMPS to delineate the posterior dissection plane easily and reproducibly. With our method, the left pancreas is resected *en bloc* and wrapped within the anterior renal fascia attached to its posterior surface.

## Methods

The surgical method was as follows.

### Clamping of the Splenic Artery

In the absence of extrapancreatic metastases, the gastrocolic ligament was excised from the colon, avoiding the gastroepiploic artery along the avascular plane juxtaposing the colon. The stomach was retracted cephalad to expose the anterior surface of the pancreas, and the short gastric vessels were divided. Subsequently, the splenocolic ligament was divided. After this step, the common hepatic artery was identified at the superior margin of the pancreas and dissected toward its origin. The origin of the splenic artery was exposed and clamped with an atraumatic bulldog clamp to reduce tumor blood flow before surgical intervention.

### Dissection of the Transverse Mesocolon

The root attachment of the transverse mesocolon leads to the retropancreatic tissues on the anterior renal fascia. To dissect the transverse mesocolon, it was retracted anteriorly and dissected widely while preserving the marginal arterial arcade. The middle colic artery and vein were ligated and divided if necessary. This procedure facilitated en bloc resection at the posterior pancreatic surface and improved visibility, making the operation easier and safer to perform.

### Dissection of the Posterior Pancreatic Surface with the Anterior Renal Fascia

**Fig. 1:**
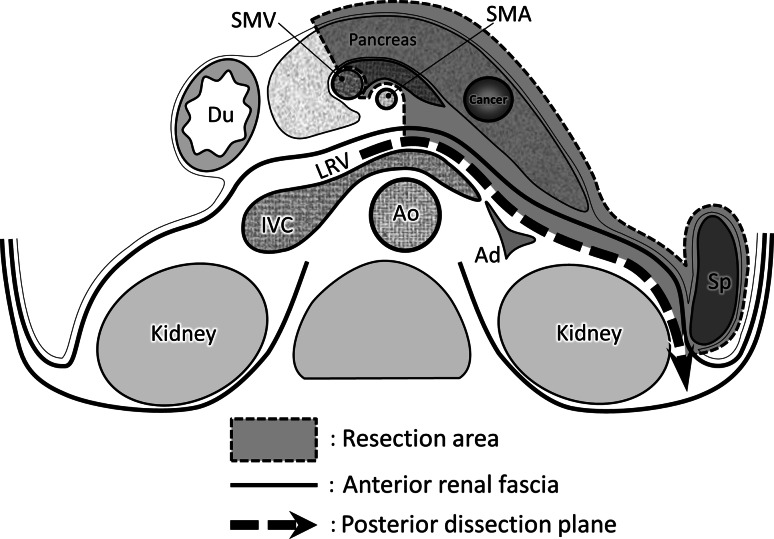
Dissection for modified RAMPS. The anterior renal fascia covers the retroperitoneal organs, including the left kidney, adrenal gland, inferior vena cava, and aorta. The pancreas is bound by, and its proper serosa fuses with, the anterior renal fascia. The posterior dissection plane for *en bloc* resections is behind this fascia. *Ad* adrenal gland, *Ao* aorta, *Du* duodenum, *IVC* inferior vena cava, *LRV* left renal vein, *RAMPS* radical antegrade modular pancreatosplenectomy, *SMA* superior mesenteric artery, *SMV* superior mesenteric vein, *Sp* spleen

**Fig. 2:**
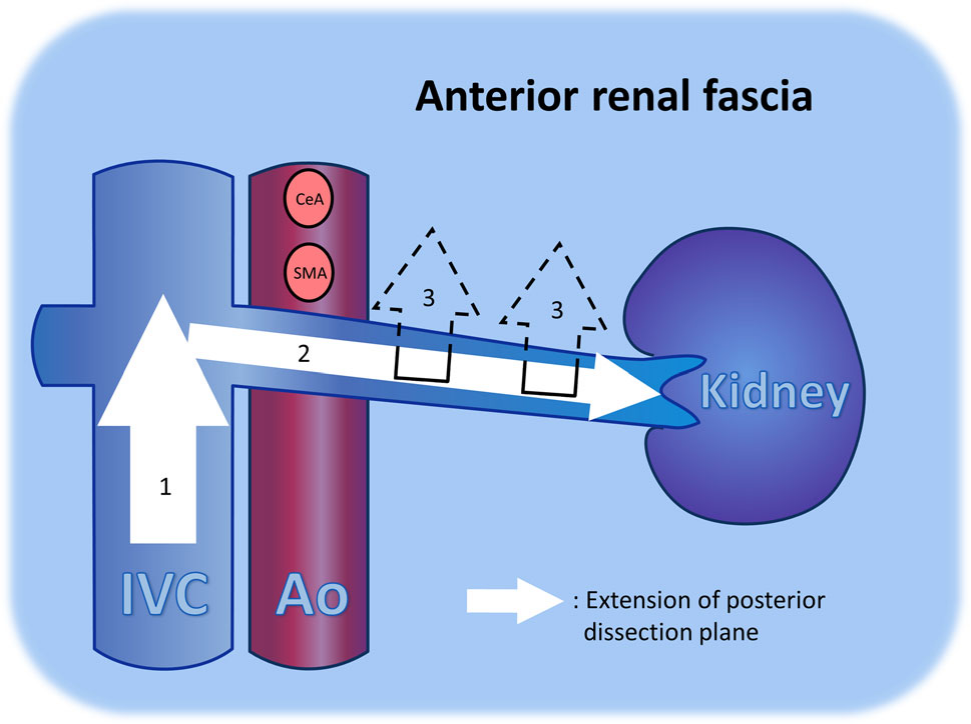
Posterior dissection plane. (*1*) The anterior renal fascia was excised on the inferior vena cava while exposing its adventitia, and this dissection plane was proceeded to the confluence portion of the left renal vein. (*2*) The dissection plane was extended left along the left renal vein toward the renal hilum. (*3*) The inferior line of dissection was extended coronal to the superior edge of the pancreas. *Ao* aorta, *IVC* inferior vena cava, *CeA* celiac axis, *SMA* superior mesenteric artery

**Fig. 3:**
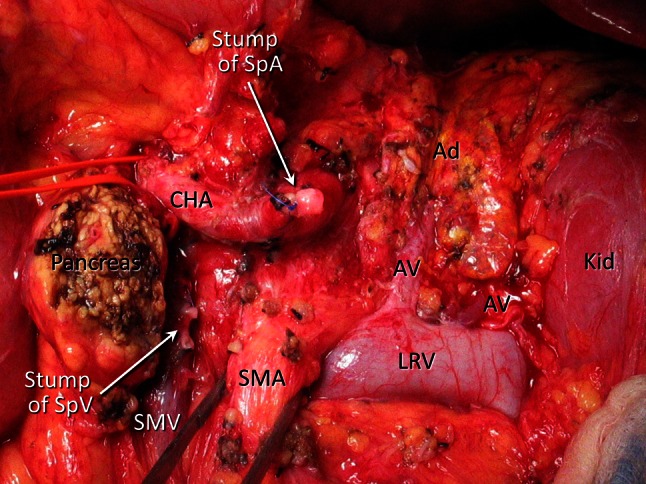
Photograph of an operation site at the end of a procedure without celiac axis resection. The left pancreas and the spleen, including the anterior renal fascia, have been resected. The left renal vein guided the posterior dissection plane, which included the anterior renal fascia. The adventitia of the renal vein was thoroughly excised. The periarterial superior mesenteric artery nerve plexus was preserved. *Ad* adrenal gland, *AV* adrenal vein, *CHA* common hepatic artery, *Kid* kidney, *LRV* left renal vein, *SMA* superior mesenteric artery, *SMV* superior mesenteric vein, *SpA* splenic artery, *SpV* splenic vein

The ligament of Treitz was resected, and the duodenum was mobilized from left to right at this location to identify the inferior vena cava (IVC). The anterior renal fascia was identified as a thin fibrous membrane covering the IVC. The posterior dissection plane was located behind this fascia (Fig. 1); therefore, the fascia was excised while exposing the IVC adventitia. The dissection plane was extended left along the left renal vein toward the renal hilum. The inferior line of dissection first ran along the left renal vein and then extended coronal to the superior edge of the pancreas (Fig. 2). The anterior surface of the adrenal veins and the adrenal gland can be used to locate this plane precisely (Fig. 3).

### Transection of the Pancreas

Initially, the lower border of the pancreas was mobilized at the planned transection line, which is usually above the superior mesenteric vein (SMV). The pancreas was transected using a scalpel. Subsequently, the splenic vein was identified and divided at its origin. The cut surface of the remaining pancreas was treated using the non-closure technique by carefully ligating the main pancreatic duct and sealing off the excised pancreatic surface by low-temperature coagulation using saline-coupled bipolar electrocauterization, as previously reported [5]. At this time, the arterial blood supply to the left pancreas was blocked completely.

### Dissection of the Celiac Node

Celiac node dissection was performed, during which, the celiac artery and its three branches were exposed, preserving the nerve plexus around the celiac artery and ganglion. At this time, the splenic artery was ligated and divided close to its origin.

### Dissection of the Retropancreatic Soft Tissues above the Superior Mesenteric Artery (SMA)

The pancreatic body lies anterior to the SMA. The soft tissue behind the pancreatic body widely contacts with the periarterial nerve plexus of the SMA. At this point, the cut end of the pancreas was retracted vertically and dissected from the retropancreatic soft tissues, including the lymph nodes. The periarterial nerve plexus of the SMA was preserved. Dissection was extended toward the previously dissected plane.

### Mobilization of the Distal Pancreas and Spleen

Dissection was then continued laterally until the phrenosplenic ligament was divided and the specimen was removed (Fig. 3). The left gastric artery was identified and saved.

When the carcinoma had spread to the nerve plexus surround the celiac axis, we performed distal pancreatectomy with en bloc celiac axis resection (DP-CAR) [6] as a more aggressive surgical treatment.

### Pathological Assessment of the Specimen

Histopathological assessments were performed by at least two pathologists. Tumors were staged according to the seventh edition of the Union Internationale Contre le Cancer (UICC) ‘tumor, node metastasis’ (TNM) classification system [7].

### Data Collection

Data on patients with adenocarcinoma of the pancreatic body or tail who were treated by modified RAMPS between June 2007 and December 2012 were entered into a prospectively collected database. Data from operation records, anesthesia records, histopathology reports, postoperative clinical course, and follow-up visits were entered into the database.

### Statistical Analysis

Pancreatic fistulae were defined using the International Study Group of Pancreatic Fistula (ISDPF) definition [8]. Overall survival was defined as the time from the operation to the date of death due to any cause. Data on survivors were censored at the date of the last contact. Median survival and the 5-year overall survival rate were determined using Kaplan–Meier survival analysis. SPSS for Microsoft^®^ Windows (SPSS version 13, Chicago, IL, USA) was used for statistical analyses.

## Results

From June 2007 to December 2012, we performed the above-described procedure on 24 patients with pancreatic invasive ductal adenocarcinoma. Patients with neuroendocrine tumors or rare malignant tumors of the pancreas were excluded from this study. No definite chemotherapy or radiotherapy was added. The patient group included 15 men and 9 women, averaging 67 years in age (Table 1).

**Table 1:** Characteristics of the study population (*n* = 24)

Characteristic	
Gender (M/F)	(15/9)
Age (year)	67 ± 9 (54–84)
Operation time (min)	387 ± 63 (274–501)
DP/DP-CAR	21/4
Bleeding (mL)	371 ± 348 (10–1500)
Concomitant resection	
Adrenal gland	5
Transverse colon	2
Portal vein (Wedge resection)	1

*DP-CAR* distal pancreatectomy with en bloc celiac axis resection, *F* female, *M* male

### Operative Procedures (Table 1)

The mean (standard deviation [SD]) operation time was 387 (63) min. The estimated blood loss was 371 (348) mL (range 10–1,500). All patients underwent resection of the left pancreas with the anterior renal fascia. Seven patients had one or two adjacent structures or organs resected. The adrenal gland was resected in five patients. Partial colectomy was required for two patients. Resection and reconstruction of the portal vein (PV) was required for one patient. DP-CAR was performed in four cases.

### Pathological Findings (Table 2)

 Tumor sizes ranged from 5 to 68 mm (mean [SD] 35 [14]). Invasion outside the pancreatic capsule into the peripancreatic soft tissues was identified in 22 of 24 (92 %) patients, i.e., 22 patients had T3 tumors and two had T2 tumors according to UICC classification [7]. The specimen lymph-node counts ranged from 10 to 51 (mean [SD] 28 [12]); the median number of nodes was 24. Of 24 patients, 13 (54 %) had between one and seven positive lymph nodes (N1) and 11 (46 %) had no positive lymph nodes (N0). All patients were classified as having M0 disease. Tumors were well differentiated, moderately differentiated, and poorly differentiated in 5, 15, and 4 patients, respectively. American Joint Commission Cancer staging was as follows: stage IA, no patients; stage IB, two patients; stage IIA, nine patients; stage IIB, 13 patients; stage III, no patients; and stage IV, no patients. A total of 23 patients had either neural or microvascular invasion. Tumor involvement of the retropancreatic tissues was confirmed in 17 cases. Two patients had a macroscopic positive margin in the transection plane at the pancreatic neck. Both had extensive direct invasion of the PV/SMV. Of 24 patients, 21 (88 %) had no residual tumor (R0).

**Table 2:** Histopathology

Characteristic	
Tumor size (max mm)	35 ± 14 (5–68)
T stage (UICC)	
Tl	0
T2	2 (8)
T3	22 (92)
T4	0
N stage (UICC)	
N0	11 (46)
Nl	13 (54)
Resected nodal number	28.0 ± 11.9 (10–51)
Metastatic nodal number	1.5 ± 1.9 (0–7)
Grading	
Gl	5 (21)
G2	15 (62)
G3	4 (17)
Stage (UICC)	
IA	0
IB	2 (8)
MA	9 (38)
MB	13 (54)
III	0
IV	0
R status	
R0	21 (88)
Rl	1 (4)
R2	2 (8)

Data are presented as mean ± SD (range) or *N* (%)

*SD* standard deviation, *UICC* Union Internationale Contre le Cancer

### Morbidity and Mortality (Table 3)

There were no postoperative (30 days) or hospital deaths. There were 11 grade B or C pancreatic fistulas (grade B, ten cases; grade C, one case). The grade C case was of a T3 tumor with involvement of the transverse colon, for which partial colectomy was performed. Failure of colonic anastomosis after partial colectomy concurred with pancreatic leakage. Reoperation for abscess drainage and colostomy was indicated. Other cases showed no intra-abdominal abscesses or sepsis. Asymptomatic hepatic infarction was a complication in two cases treated with DP-CAR.

**Table 3:** Morbidity and mortality

Surgical morbidity	
Pancreatic fistula (grade B, C)	11 (46)
Liver infarction	1 (4)
Portal thrombosis	1(4)
Failure of anastomosis (colon–colon)	1 (4)
Hemorrhage	0
Gastric ulcer	0
Perioperative mortality	0

Data are presented as *N* (%)

### Patient Survival Rates

**Fig. 4:**
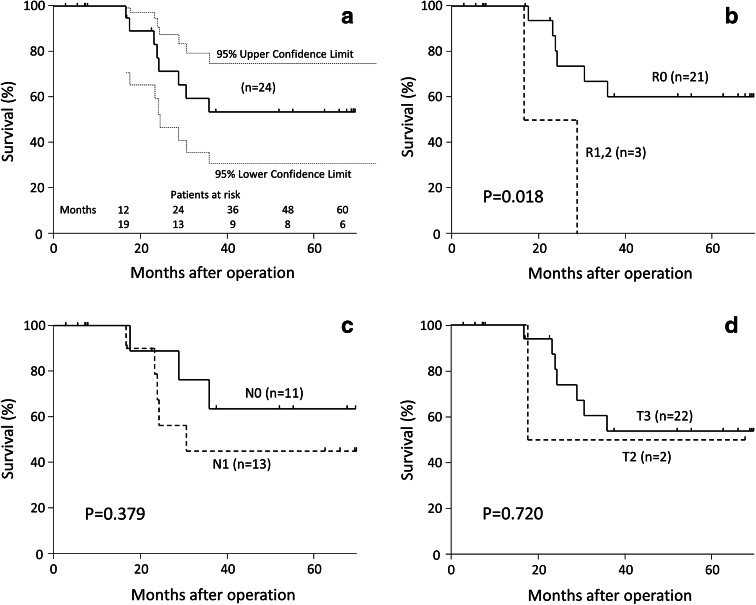
Kaplan–Meier survival estimations. **a** A total of 24 patients who underwent modified RAMPS for treating ductal adenocarcinoma of the left pancreas. Survival data were censored at 70 months, the latest time point at which >10 % of the 24 patients could be followed. Within the 70-month follow-up period, hatch marks indicate the times corresponding to censored patients. **b** Overall survival curve according to patients’ histopathological status. **c** Overall survival curve according to the status of lymph-node metastases. **d** Overall survival curve according to T factor status. *RAMPS* radical antegrade modular pancreatosplenectomy

Mean and median follow-up times for surviving patients were 27 and 52 months, respectively; 12 patients survived without evidence of disease. The overall survival curve is shown in Fig. 4a. The 5-year overall survival rate was 53 % (95 % confidence interval [CI] 31–75). Six patients survived for over 5 years without recurrence. Surgical margins were histologically clear (R0 resection) in 21 patients (88 %). The estimated 5-year survival rate for R0 patients was 60 % (95 % CI 35–81, Fig. 4b). Recurrence after R0 resection was detected in ten patients. Local recurrence was found in three patients, liver metastases in six, and peritoneal dissemination in three. Local recurrence sites were the area surrounding the celiac axis, the area surrounding the common hepatic artery, and lymph nodes embedded in the hepatoduodenal ligament. The overall survival curve reflecting the status of lymph-node metastases is shown in Fig. 4c. For N1 cases, median survival was 24 months, and the 5-year overall survival rate was 45 % (95 % CI 18–75). The overall survival curve reflecting the T factor status is shown in Fig. 4d. The 5-year survival rate was 54 % (95 % CI 30–76) for T3 cases.

## Discussion

During the past decade, a number of high-volume hospitals have reported significant improvements in postoperative survival rates following distal pancreatectomy in pancreatic cancer patients. However, cancer recurrence or low survival rates following surgical resection of the body or tail of the pancreas has commonly been reported. Local relapse or liver metastases are the most common complications despite great efforts toward recurrence prevention. In our previous study of 74 patients with resected ductal carcinoma of the body or tail of the pancreas, only five patients (7 %) had microscopically curative resections [9]. Further, only three patients with microscopically curative resections survived beyond 3 years.

Positive resection margins after surgery for pancreatic adenocarcinoma are reportedly an independent negative survival prognosticator [10–13]. Therefore, achieving negative surgical margins is essential for patient survival. Cancer propagation to peripancreatic soft tissues should be delineated precisely and resected with adequate margins. Recently, distal pancreatectomy with extended dissection of retroperitoneal structures and extended lymphadenectomy has been shown to improve operation outcomes [6]. Four prospective randomized trials for treating pancreatic head cancers compared standard pancreaticoduodenectomy and pancreaticoduodenectomy plus extended lymphadenectomy and reported no improvement with the more radical operation [14–17]. These studies indicate that extended lymphadenectomy does not necessarily contribute to favorable survival outcomes. Therefore, standard regional lymphadenectomy with negative surgical margins for patients with carcinoma of the left pancreas are paramount.

Because the pancreas body and tail are thin structures, carcinomas can easily spread beyond pancreatic parenchyma. The left pancreas attaches closely superior to retroperitoneal organs, including the kidney, adrenal gland, and IVC, which are demarcated with the anterior renal fascia [18]. Although the pancreatic carcinoma easily infiltrates near to the anterior renal fascia beyond parenchyma, involvement of retroperitoneal organs beyond the anterior renal fascia is uncommon [19]. In en bloc resections of the left pancreas, the anterior renal fascia is an important structure. Moreover, the regional lymphatic vessels, which surround the pancreatic parenchyma along its superior and inferior edges, exist above the anterior renal fascia. Therefore, en bloc resection with the anterior renal fascia facilitates entire removal of accompanying lymphatics of the left pancreas. This thin microscopic fascia is difficult to recognize intraoperatively, except for the fascia surrounding the IVC and left renal vein. The posterior dissection plane used in our modified procedure starts on the adventitia of these veins and advances to upper edge of the pancreas.

Pancreatic lymphatic vessels have been reviewed in detail using macroscopic dissections by Deki and Sato [20]. Accordingly, the left pancreas has been divided into three parts: the median subsegment of the body, the left subsegment of the body, and the tail. The former corresponds to the ‘body’ according to the definition by the American Joint Committee on Cancer (AJCC) [21]. The two latter segments correspond to the ‘tail’ according to the AJCC definition. Regional lymphatics of both the tail and the left subsegment of the body are bound by the splenic artery and vein and the pancreatica magna artery. In the center, they are bound by the origin of the splenic artery. Regional lymphatics of the median subsegment of the body extend to the trunk of the common hepatic artery and the left gastric artery. The segmental AJCC definitions of ‘body’ and ‘tail’ are more logical for the distribution of lymphatics than the definitions by Deki and Sato. Therefore, resection of lymphatics is determined by whether the pancreatic body has been infiltrated by the malignant tumor. Regional lymphatics are dissected *en bloc* from the origin of the splenic artery to the splenic hilum if the tumor is confined to the tail. In contrast, additional regional lymphatics are to be dissected *en bloc* around the common hepatic artery and celiac axis if the tumor extends to the body. Theoretically, DP-CAR is a valid procedure for regional lymphatic dissection in pancreatic body carcinoma [6]. However, DP-CAR was performed in only a few cases in our series, and multicenter trials are necessary for thorough analysis.

In our study, modified RAMPS was effective in advanced cases with tumors extending beyond the pancreas or with concomitant nodal involvement. Our results highlight the advantages of our procedure in achieving negative surgical margins at peripancreatic soft tissues by *en bloc* resection of the lymphatic basin of the carcinoma of the left pancreas. The anterior renal fascia, which is resected through the posterior dissection plane, is also important in combatting the carcinoma [19]. This fascia is thin but clearly visible on the left renal vein in all cases. The left renal vein is present behind the lower edge of the left pancreas; therefore, the left renal vein is a landmark for determining the posterior dissection plane, including the anterior renal fascia.

Originally, RAMPS was established by Strasberg et al. [3, 4, 22] based on the following three principles: N1 lymph node dissection, modular setting of the posterior plane of dissection optimized to achieve negative posterior margins, and right-to-left dissection to achieve early vascular control. They specified that the depth of the posterior dissection should always lie behind the anterior renal fascia and that the plane of the posterior margin should lie behind the anterior renal fascia while dissected onto the left renal vein. The left renal vein is an important guide for posterior dissection including the anterior renal fascia. Using the Strasberg method, the left renal vein is exposed where it passes behind the superior mesenteric artery and in front of the aorta. The Kocher maneuver was reported to facilitate identification of the renal vein. Rosso et al.[23] stated that the wide mobilization of the pancreas head and the duodenum from the inferior edge of the Winslow foramen, including the right colon and the mesentery, is useful for good vision during the operation. Our procedure differed from their method with respect to how the left renal vein was identified and how the posterior dissection plane was extended behind the anterior renal fascia. We first approached the IVC after mobilizing the fourth and third duodenal portions from left to right and dissected upward along the posterior plane to the anterior renal fascia to identify the confluent point of the left renal vein. The Kocher maneuver, which is performed for mobilizing the head of the pancreas, is not necessary for our method. For pancreatic carcinoma involving the PV or SMV, the Kocher maneuver would be useful to perform reconstruction after sleeve resection. In the original procedure, the short gastric vessels were divided at the last operational stage before specimen removal. However, because the short gastric arteries constitute the major arterial supply to the spleen after division of the splenic artery in the Warshaw procedure [24], we shut out the short gastric vessels and the splenic artery at an early stage. Shutting out these two arterial supplies constitutes ‘early vascular control’ for pancreas or spleen resection.

Two of our study cases showed focal tumor infiltration to the adrenal glands beyond the anterior renal fascia; these were diagnosed preoperatively using dynamic multi-detector row computed tomography (MDCT). Planned resections for achieving negative margins were performed. MDCT is useful in diagnosing whether the carcinoma has infiltrated beyond the fascia.

Recently, the use of RAMPS with venous resection for en bloc resection of pancreatic cancer involving the PV or SMV was reported by Rosso et al. [23]. In one of our study cases, wedge resection of the PV was performed. Unfortunately, in this patient, the margin was positive in the transection plane at the pancreatic neck. This method would be feasible and appropriate for patients with PV or SMV involvement, so long as R0 resection is possible. In our experience, the additional involvement of the hepatic artery or celiac artery is problematic in these patients.

The confidence limits were wide, as the number of cases were small, but all cases were followed at our institute and there were no ‘outcome unknown’ cases in censored cases. Three of our study cases showed positive resection margins. One patient had a positive intraparenchymal margin, and posterior margins were positive in two cases. In summary, 21 of 24 (88 %) patients showed negative margins and 22 of 24 (92 %) patients showed negative tangential margins, results that were as favorable as those reported by Mitchem et al. [22].

## Conclusion

This modification of RAMPS allows us to perform *en bloc* resections and achieve clear posterior margins and adequate dissection of regional lymphatics for treating left pancreatic carcinomas.
